# Septic Arthritis of the Right Wrist Due to Mycobacterium avium Complex in an Immunocompetent Patient

**DOI:** 10.7759/cureus.17129

**Published:** 2021-08-12

**Authors:** Bibek Saha, Kurtis Young, Melissa Kahili-Heede, Sian Yik Lim

**Affiliations:** 1 Medicine, John A. Burns School of Medicine, University of Hawaii at Manoa, Honolulu, USA; 2 Rheumatology, Pali Momi Medical Center, Aiea, USA

**Keywords:** mycobacterium avium intracellulare, mycobacterium avium complex, septic arthritis, case report, non-tuberculous mycobacterium

## Abstract

Septic arthritis due to *Mycobacterium avium* complex (MAC) is extremely rare. While MAC infection is classically associated with HIV/AIDS and immunosuppressed states, it may occur in immunocompetent individuals. We report a case of MAC septic arthritis of a native wrist joint in an immunocompetent host. The diagnosis of septic arthritis due to MAC is commonly delayed and initially misdiagnosed, warranting a high level of suspicion to make an accurate and timely diagnosis. Suspecting the diagnosis when there are atypical features present in the clinical history may be crucial in identifying affected patients.

## Introduction

*Mycobacterium avium* complex (MAC), classified as non-tuberculous *Mycobacterium*, is now a well-documented cause of infectious disease in humans [1]. Specifically, MAC presents as disseminated disease in patients with HIV/AIDS and as pulmonary disease in patients with chronic lung conditions [1]. Extrapulmonary disease is less frequent and can include lymph node infections in children, as well as infections of the skin, soft tissue, and musculoskeletal system [1]. Septic arthritis due to MAC, however, is extremely rare. Here we present a case of MAC septic arthritis of a native joint in an immunocompetent patient. This article was previously presented as an oral presentation at the 2021 Virtual Biomedical Sciences & Health Disparities Symposium on April 15, 2021.

## Case presentation

An 82-year-old woman presented to the rheumatology office with right wrist swelling for nine months. Seven months before presenting at our clinic, she initially visited her primary care doctor, who treated her for suspected gout. X-ray of the right wrist during that visit demonstrated degenerative changes. Labs obtained at that time showed an elevated anti-nuclear antibody (1:80), but rheumatoid factor, anti-cyclic citrullinated peptide antibody, anti-smooth muscle (Sm), anti-Sm/ribonucleoprotein, and anti-dsDNA were negative. C-reactive protein was less than 5.0 mg/L, and her uric acid level was 6.8 mg/dL.

She was treated with a 10-day course of oral prednisone 20 mg daily for seven days with some improvement of her right wrist pain and swelling. However, her symptoms recurred after completing her prednisone course. She was subsequently treated with three oral prednisone courses with only mild improvement of her symptoms, but her symptoms never entirely resolved while on prednisone. She visited urgent care, where she was prescribed sulfamethoxazole-trimethoprim (TMP-SMX) 80-160 mg twice a day for 10 days for possible cellulitis. There was a significant improvement with TMP-SMX treatment, but the wrist pain recurred after completing the antibiotic course.

The patient was then referred for rheumatology evaluation. She was afebrile on presentation, and tenderness, swelling, and warmth of the right wrist were appreciated. There was no swelling or tenderness in any other joint. Her lung, cardiovascular, and abdominal examination were all unremarkable. Her past medical history was significant for hypertension, hyperlipidemia, osteoporosis but not diabetes. Her medications included alendronate 70 mg weekly, losartan 50 mg daily, metoprolol 50 mg daily, and lovastatin 20 mg daily. Labs were unremarkable, except for a C-reactive protein of 7.7 mg/dL. Aspiration of the joint was attempted, but no fluid was obtained from the right wrist.

The lack of response to prednisone treatment and improvement with TMP-SMX therapy led to the suspicion of chronic infection. Urgent referral to orthopedic surgery was made. Orthopedic surgery performed synovial biopsy via a dorsal approach. Rice bodies were noted intraoperatively. Histopathology of the synovial biopsy showed chronic inflammatory findings, with fibrinoid necrosis but no granulomas.

No microorganisms were seen on the gram stain on the initial synovial biopsy, and no acid-fast bacilli were detected on the acid-fast bacilli smear. No growth was noted on the culture at three days. One week later, culture by Mycobacteria Growth Indicator Tube method returned positive for acid-fast bacilli. Additionally, the MAC DNA probe was positive, while the *Mycobacterium tuberculosis* complex DNA probe was negative. Susceptibility testing of the MAC showed resistance to amikacin, but sensitivity to clarithromycin, linezolid, and moxifloxacin. The bacterial culture at 30 days was negative.

The patient was treated with a combination of rifampin 300 mg daily, ethambutol 400 mg daily, and azithromycin 250 mg daily for six months and noted significant improvement of her symptoms. Her right wrist pain and swelling resolved after treatment.

## Discussion

We describe a case of right wrist septic arthritis due to MAC in an immunocompetent older female. Our case highlights the need for a high level of suspicion for the possibility of MAC septic arthritis presenting as chronic refractory arthritis, even in immunocompetent individuals. Prior case reports, and ours, have demonstrated that septic arthritis due to MAC is frequently not suspected at initial evaluation [2,3]. That is, due to the insidious nature of presentation, the diagnosis may be delayed for many years with misdiagnosis being common [2-4]. Suspecting MAC septic arthritis when there are atypical features present in the clinical history may be key in achieving an accurate and timely diagnosis.

Many cases in the literature have reported septic arthritis due to MAC in patients with a weakened immune system [4,5]. These include patients with autoimmune disease on immunosuppressive treatment [4,6]. Specifically, cases of MAC septic arthritis have been reported in patients with rheumatoid arthritis, scleroderma, and dermatomyositis, and on medications such as prednisolone, azathioprine, and infliximab [4]. Several cases have reported MAC septic arthritis occurring in patients infected with HIV [5,7,8]. Similarly, MAC septic arthritis has also been reported in patients with intrinsic immunodeficiency [3]. However, MAC septic arthritis has been reported previously in immunocompetent individuals, with no apparent risk factors, similar to our case report. Vrettos et al. reported a case of MAC in an immunocompetent host affecting the sternoclavicular joint [9]. Similarly, Parperis et al. reported a case of destructive monoarthritis in the fourth proximal interphalangeal joint due to MAC, also in an immunocompetent host [10]. Both patients had no history of autoimmune disease, were not taking any immunosuppressive medications, and did not have HIV or any intrinsic immunodeficiency. Additionally, no risk factors such as intraarticular steroid injections or prior trauma were noted.

The diagnosis of MAC septic arthritis can be challenging. Due to the insidious nature of the disease process, the low prevalence of the condition, and current modalities available in establishing the diagnosis, accurate diagnosis is frequently delayed [3]. Although diagnosis can be established through mycobacterial culture, or direct nucleic acid amplification of synovial fluid obtained from arthrocentesis, many cases of MAC septic arthritis require obtaining synovial tissue biopsy for culture and testing. In a recent study of 33 cases reported in the literature, approximately 50% of cases required a biopsy [3].

In our case, lack of response to prednisone and improvement on TMP-SMX treatment were clues that led to appropriate workup and diagnosis. Recognizing atypical features may be important in establishing an accurate diagnosis. For example, Bridges et al. reported cases of MAC septic arthritis in a dermatomyositis patient, and a scleroderma patient on immunosuppression. Both patients presented with oligo- or monoarthritis with significant synovitis, a feature not common in these autoimmune pathologies. The authors suggested that the diagnosis of *Mycobacterium intracellulare* be considered in such presentations [4]. Kanik et al. presented a case of polyarthritis, initially misdiagnosed as seronegative rheumatoid arthritis. The patient was subsequently diagnosed with MAC septic arthritis, the diagnosis suspected after the patient’s condition had worsened on disease-modifying anti-rheumatic drug treatment [11].

The clinical course of MAC septic arthritis is variable. In general, the clinical course is considered insidious, chronic, and indolent without joint or bone destruction [12]. However, disseminated and erosive cases have been reported [8,13]. Treatment guidelines have been published by the American Thoracic Society and Infectious Disease Society of America [14]. The treatment usually consists of surgery plus anti-mycobacterial treatment, or anti-mycobacterial treatment only. For septic arthritis due to MAC, combined surgery with anti-mycobacterial treatment is recommended, which may be associated with improved outcomes as compared with anti-mycobacterial treatment alone [3]. An empiric regimen of macrolide-based multidrug treatment is initiated with subsequent treatment guided by cultures and sensitivities for 12 months or potentially even longer for severe infections.

## Conclusions

In summary, we present a case of MAC septic arthritis in an immunocompetent patient. Recognizing risk factors and suspecting the diagnosis in certain atypical clinical situations are important for accurate and timely diagnosis. More research is warranted with regard to the clinical course of MAC septic arthritis.
